# Selection of Statistical Thresholds in Graphical Models

**DOI:** 10.1155/2009/878013

**Published:** 2010-03-04

**Authors:** Anthony Almudevar

**Affiliations:** 1 Department of Biostatistics and Computational Biology, University of Rochester, 601 Elmwood Avenue, Box 630, Rochester, NY 14642, USA

## Abstract

Reconstruction of gene regulatory networks based on experimental data usually relies on statistical evidence, necessitating the choice of a statistical threshold which defines a significant biological effect. Approaches to this problem found in the literature range from rigorous multiple testing procedures to ad hoc *P*-value cut-off points. However, when the data implies graphical structure, it should be possible to exploit this feature in the threshold selection process. In this article we propose a procedure based on this principle. Using coding theory we devise a measure of graphical structure, for example, highly connected nodes or chain structure. The measure for a particular graph can be compared to that of a random graph and structure inferred on that basis. By varying the statistical threshold the maximum deviation from random structure can be estimated, and the threshold is then chosen on that basis. A global test for graph structure follows naturally.

## 1. Introduction

The reconstruction of gene regulatory networks using gene expression data has become an important computational tool in systems biology. A relationship among a set of genes can be established either by measuring the effect of the experimental perturbation of one or more selected genes on the remaining genes or from the use of measures of coexpression from observational data. The data is then incorporated into a suitable mathematical model of gene regulation. Such models vary in level of detail, but most are based on a *gene graph*, in which nodes represent individual genes, while edges between nodes indicate a regulatory relationship. 

One important issue that arises is the variability of the data due to biological and technological sources. This leads to imperfect resolution of gene relationships and the need for principled statistical methodology with which to assign statistical significance to any inferred feature. 

In many models, the existence or absence of an edge in the gene graph is resolved by a statistical hypothesis test. A natural first step is the ranking of potential edges based on the strength of the statistical evidence for the existence of the implied regulatory relationship. The intuitive approach is to construct a graph consisting of the highest ranking edges, defined by a -value threshold. The choice of threshold may be ad hoc, typically a conservative significance level such as 0.01. A more rigorous approach is to select the threshold using principles of multiple hypothesis testing (see, e.g., [1]), which may yield an estimate of the error rates of edge classification. 

There is a fundamental drawback to this approach, in that the lack of statistical evidence of a regulatory relationship may be as much a consequence of small sample size as of biological fact. Under this scenario, we note that selection of a -value threshold  generates a graph of, say,  edges, with  increasing in . Under a null hypothesis of no regulatory structure, -values are randomly ranked, hence edges will be distributed uniformly, whereas the edges of a true regulatory network will posses structure unlikely to arise by chance. Formulated in terms of statistical hypothesis tests, it should be possible to exploit this evidence in order to make a more informative choice of . This article proposes a method to accomplish this goal.

## 2. Problem Formulation

The proposed algorithm is intended to be part of the following type of analysis based on gene expression data for  genes.

(S1) Collect gene perturbation data from  experiments coupled with control data. For simplicity, assume that experiment  is a single perturbation of gene .

(S2) Construct an  matrix , in which element  is a measure of the statistical evidence that perturbation of gene  changes the expression level of gene . This defines *data matrix*.

(S3) Use the methodology proposed here to determine a -value threshold .

(S4) Conclude that perturbing gene  causes a change in gene  if and only if .

(S5) The perturbation responses implied by step (S4) may now be used to fit, for example, a Boolean network as in [2–4].

We assume in (S1)-(S2) that the matrix  is *balanced* in the sense that row  and column  refer to the same gene. The methods proposed here do not rely on this assumption, although a formal treatment of the general case will be deferred to future work. Typically,  will be a -value from a two-sample hypothesis test comparing the expression levels of genes  obtained from cells subject to an experimental perturbation of gene  to those obtained from control (unperturbed) cells. In this case small values of  are interpreted as evidence for the existence of directed edge . We adopt this convention below. 

It will be useful to introduce some definitions of directed gene graphs (see [5]). We say gene *regulates* gene  if the gene expression level of  directly influences that of gene . This is distinct from *transitive regulation*, in which expression levels of one gene affect another only through intermediary genes. For example, if  regulates  and  regulates , then  and  are in a transitive regulatory relationship (that would not exist without ). In an *accessibility graph* edge  exists if  regulates or transitively regulates . In contrast, in an *adjacency graph* an edge from  to  exists only if  regulates . An adjacency graph can be constructed as a parsimonious representation of an accessibility graph ([5–7]). It should be noted that a regulatory relationship implied by a graphical model is relative only to those genes included and does not rule out the existence of intermediary genes not observed. 

Step (S3) will be based on the following idea. Data matrix  can generate an estimated accessibility graph  by constructing an edge  if and only if . While this is a crude form of network model, we may still expect  to contain interesting and measurable structure, provided that  is efficiently chosen. Our intention is to use this structure to guide the choice of . The set of edges in  is then used to construct a more detailed model, as in step (S5). 

Consider a hierarchical sequence of graphs  obtained by successively adding edges in increasing order of their -values. If the data is dominated by statistical noise, we may expect elements of the sequence to consist of random graphs generated by uniform distributions of a fixed number of edges, known as the Erdös-Renyi random graph model (see, e.g., [8]). Actual cellular networks are believed to conform more closely to the power-law model, where the likelihood that a randomly chosen node has  interactions is proportional to  where  (see [9]). We may also expect more chain structure (longer paths) than would occur by chance. This would allow statistical identification of cellular network structure, which can provide auxiliary information for the selection of  beyond what is normally available using standard multiple hypothesis testing methods.

### 2.1. Conditional Hypothesis Tests

The required elements of our procedure are (i) a data matrix  (steps (S1)-(S2)), (ii) a graph score  which is sensitive to general graphical structure, and (iii) a distributional model  for generating graphs under the null hypothesis of no regulatory relationships. In the following development smaller values of  imply greater structure.

#### 2.1.1. Notational Conventions

We will adopt the following notation. Assume that  is fixed. First, let  be the set of all increasing sequences of positive integers  for which . Then let  be the set of all -dimensional vectors of nonnegative integers (which we refer to as *count vectors*). Let  denote the sum of the elements of any . A sequence of vectors  from , written , is *increasing* if  for all , , and if . Let  be the set of all such increasing count vector sequences. 

The set of all order  labelled graphs is denoted by . Let  be the subset of graphs with  edges, and for any  let  be the subset of graphs containing  edges with parent . Let ,  be the respective subsets which exclude all edges from edge set . A sequence of graphs  from  is called *increasing* if  is a subgraph of , . We say that an increasing graph sequence   *conforms* to index sequence  and set  if , for all .

#### 2.1.2. Data Matrix

Suppose that we are given an  data matrix  of -values as described in (S1)–(S5). An edge  may be ruled out by setting . We will refer to such an edge as a *void* edge, with corresponding *void* matrix element. For example, this should occur when the data cannot predict self-regulation implied by edges . A missing value in  may also represent a void edge. 

Let  be the sequence of all unique values represented as elements of . The value of  varies according to the number of ties as well as the number of void elements. We need to define a system of counts generated by . Set(1)

where  when event  occurs and is zero otherwise. We then define the sequence , where . This sequence is increasing, and consecutive graphs may increase by more than one edge. We refer to the system of counts  and ,  which can be interpreted as random objects in sample spaces , , respectively. The sequence  corresponds to the number of edges of the graphs in ; that is,  is the number of edges in . Similarly,  corresponds to the number of edges decomposed by parent node in ; that is,  is the number of edges in  with parent node .

#### 2.1.3. Conditional Inference

Under the simplest null hypothesis of no regulatory structure perturbation conditions are indistinguishable from the control, in which case the -values of  are uniformly distributed. A number of considerations then need to be made. The uniform distribution assumption depends on a correct characterization of the sampling distribution, which is often problematic in gene expression assays. In addition, when empirical methods (permutation or bootstrap methods) are used to estimate -values, ties may result which affect graph ordering. Finally, the definition of a null model relies on the independence structure of the data, which must be carefully characterized. Conditional procedures permit the development of tests which do not depend on problematic model identification, and have been extensively used in other applications in statistical genetics. 

We will now develop two null models. A conditional inference procedure is defined by data , a composite null hypothesis  concerning , a test statistic , an ancillary statistic , such that the distribution of  conditional on  can be characterized, and is the same for all distributions described by .

#### 2.1.4. Null Model 1 (Elementwise Exchangeability)

Recall that a multivariate distribution is *exchangeable* if it is invariant under any permutation of its coordinates. This includes  distributions, but also those with identical marginal distributions and permutation invariant dependence structure.

For any  let  be all graphs in  for which  is a sub graph.

Definition 1.

For void edges  and sequence , a random sequence of graphs  possesses a null distribution  if  is uniformly distributed on  and if , conditional on , is uniformly distributed on , for all .

The sequence  forms a Markov process in which  is obtained from  by adding  edges to  at random, excluding . We then define the null hypothesis: 

() The distribution of the nonvoid elements of  is exchangeable.

This leads to the following lemma. 

Lemma 1.

Under hypothesis  the distribution of  conditional on  is given by .

Proof.

Suppose  for some . For  suppose  and . We have the set equality (2)

Let , and suppose . We may define a permutation operator  on the nonvoid elements of  such that the elements associated with  are mapped onto the elements associated with , with element associated with  mapped into themselves. This implies that the elements not associated with  are mapped into the elements not associated with . The quantity  is permutation invariant, and by construction  so from (2) we have (3)

By the exchangeability assumption  and  have identical distributions, giving (4)

The argument can be adapted to verify that , conditional on  is uniformly disributed on . By combining the above equalities the proof follows. 

In the simplest case, the null hypothesis predicts uniformly distributed and independent -values among nonvoid elements of . In this case by Lemma 1   conditioned on  has distribution . If the marginal distributions are continuous, then the probability of ties is zero, and with probability 1 the elements of  increment by one until the void elements are reached. When distributions are discrete ties, are possible and  can be determined directly from the data. It is important to note that the actual marginal distribution of the elements is not important, which is a considerable advantage when null distributions are difficult to estimate accurately. 

The testing procedure proposed here is based on simulated sampling from . There are two straightforward ways to do this. First, let  be a random matrix obtained by a random permutation of the nonvoid elements of . We have already argued that . We also note that the distribution of nonvoid elements of  is exchangeable, hence by Lemma 1   has distribution . Alternatively, suppose  is any random matrix with continuously distributed  nonvoid elements. Given any index sequence , we can define a sequence of graphs . It is easily verified that  has distribution .

#### 2.1.5. Null Model 2 (Within Column Exchangeability)

The use of  as a null distribution rests on the assumption of elementwise exchangeability. A number of commonly encountered conditions may require alternative assumptions. For example, the columns of  may be derived from data obtained from a single high throughput assay. In this case, the columns may be independent, but not identically distributed. Furthermore, normalization procedures and other slide specific factors may affect any independence assumptions within a column. We therefore develop an alternative null model based on within column exchangeability, which is accomplished by conditioning on .

Definition 2.

Suppose that we are given void edges  and an increasing count vector sequence , . A random sequence of graphs  possesses a null distribution  if  is uniformly distributed on , and if  conditional on  is uniformly distributed on  for all .

Then define our second null hypothesis:

() The columns of  have an exchangeable distribution among nonvoid elements, and are mutually independent.

This leads to the following lemma. 

Lemma 2.

Under hypothesis  the distribution of  conditional on  is given by .

Proof.

The argument in Lemma 1 may be directly adapted by using only permutations  which map any element into its original column.

Following the permutation procedure used to simulate , we can simulate  using independent within column permutations of , resulting in . By Lemma 2,  possesses distribution . We note that by construction a graph sequence sampled from  also conforms to .

### 2.2. Hypothesis Test Algorithm

Suppose that we have a sample of graphs  from a distribution , which in turn defines a random variable  with distribution , where  is distributed as . If  is a null distribution representing graphs with no significant structure, then the location of  in the lower tail of  is evidence of significant structure within . 

We will assume that when null hypothesis  or  does not hold, this violation is due to the existence of a true graph . In this case, all elements of  conform to the null hypothesis except for any  for which , which are assumed to have smaller means than would be implied under the null distribution. 

We therefore define statistics:(5)

where  and  are the sample mean and standard deviation of sample . Then  is the estimated -value for a test against a null hypothesis that  is sampled from , and  is the associated -score. 

Now suppose that we are given , with , . We may generate a random sample from either  or , say . Set , from which we extract sample  so that when  is a random sample from  or ,  is a uniformly distributed random sample from  or , respectively. This leads to the two sequences of statistics:(6)

These sequences then form measures of the deviation of  which can be used to accomplish two tasks. First, we conjecture that the minimum point of these sequences will define a useful threshold , that is, a point in the sequence  below which most edges are true positives (a selected edge in true graph ), and above which additional edges are primarily false positives (a selected edge not in true graph ). Second, by generating further replications, we can estimate a global significance level for the presence of network structure. As will be discussed below, examining the entire range of the sequence  may be problematic, and so it may be truncated. Let , where . Then consider the truncated sequences:(7)

Thus, all graphs of order  or less are considered. We first devise a statistic  which measures statistical differences of  from the sample . We then generate an additional set of  null replications from the null distribution, denoted by . An empirical distribution is formed from the sample , from which a significance level for statistic  is directly obtainable. This represents the desired global significance level. We consider the four choices:(8)

We now summarize the proposed algorithm.

Algorithm 2.2 A.

(1) Construct hierarchical graph sequence  for data matrix .

(2) Generate reference sample  from  replications of null model  or .

(3) Identify threshold  as the minimum point of the sequence  (or alternatively of ).

(4) Generate a new reference sample  from  replications of the null model.

(5) Calculate statistic , and determine its quantile position among replications , *…* ,. This gives the global significance level for the presence of graphical structure in the network.

The Algorithm A depends on a score  which is sensitive to general forms of regularity. This is discussed in the next section.

## 3. Information-Based Scoring for Directed Graphs

Information theoretic methods are becoming increasingly important in bioinformatics (see, e.g., [10]) and have been recently used in various graphical modelling applications. Recent examples include [2–4, 11, 12]. This is generally done using the *minimum description length* (MDL) principle, [13–15], which is a general method of inductive inference based on the idea that a model's goodness of fit can be objectively measured by estimating the amount of data compression that it permits. The work proposed here is not formally an application of these methods but does share an interest in coding techniques for graphs.

### 3.1. Coding-Directed Graphs

The present objective is to devise a coding algorithm for a directed graph  using efficient coding principles [16]. The object to be coded is first reduced to a list of elements in a predetermined order (letters of a text or pixels of an image). Each element is coded separately into a codeword of binary digits, which are then concatenated to form one single binary string. It is important to ensure that each distinct object is converted to a unique code, and this may be done by ensuring that the codewords possess the *prefix property*; that is, no codeword is a prefix of another codeword. The simplest such code is the *uniform code*. If an element to be coded is one of  types, then each type can be uniquely assigned a binary string of  bits, and any concatenation of uniform codes can be uniquely decoded. In the following development we will forgo the practice of rounding up to the next integer, since in the context of inference it is more intuitive for the code length to be a strictly increasing function of . 

In order to code a nonnegative integer using a uniform code we would have to specify an upper bound , giving  types, and so a codeword length of  for each integer. If we expect most integers to be significantly smaller than , this would be inefficient. We will therefore make use of a *universal code* proposed in [17]. One segment of the code consists of a binary representation of the integer, with no leading 0's. The code is prefixed by a string consisting of 0's equal in length to the binary string followed by a 1. Thus, , , , and so on. In general, we will have code length  when , and  for . This code is a prefix code, with the advantage that no upper bound need be specified, and it will be more efficient when smaller integer values are expected to be most prevalent. In the work which follows, we omit the rounding operation, and so accept the approximate code length of  as(9)

Again, it is more natural that  be strictly increasing. 

A directed order  graph may be represented as an  0-1 *adjacency graph* (the class of such matrices is denoted by ). An edge from node  to  is indicated by a 1 entry for row  and column . Such a matrix may be completely represented by an ordered list of  subsets of , in which the th subset represents the entries of row  equaling 1. The graph itself may therefore be coded as a concatenation of  codewords representing the subsets. We assume that the value of  is available to the decoder. 

To code a subset, a uniform code may used, so that any subset from  labels would be coded using  bits. However, in the applications considered here, it is often expected that the size of the subset is considerably smaller than . An alternative strategy is to first specify the size  of the subset and then apply a uniform code to represent all subsets of that size. This involves concatenating a codeword for  (using the universal integer code) and a codeword for the subset (using a uniform code for  possible subsets). A subset of size  from  objects will then be assigned a code length of(10)

We refer to this code as an *size indexed* code, in contrast to a uniform code. A code for matrix  is then easily constructed by concatenating codewords for each row subset, giving code length(11)

where  is the number of 1 entries in row . This code is similar to the one proposed in [11] but assumes that  bits are used to code , as required by a uniform code on  integers. 

There will be some advantage to considering a modification to . If only a relatively small subset of nodes possess edges, then we may instead code a submatrix of . Let  be the set of nodes which are part of at least one edge. Possibly, , in which case it may be advantageous to code only the  submatrix of . But we would also need to code  itself. This object may be converted to codewords using the size indexed code and will appear in the code as a header, followed by the submatrix coded as described above. Thus, the code length for the  submatrix is(12)

### 3.2. Properties of Graph Codes

We now examine the properties of the scores. In Algorithm A comparisons of graphs will be between those with equal numbers of edges. We will consider an asymptotic scenario in which the size of the largest subset is bounded by , with . Applying Lemma  of [18] we may write (13)

where  is the number of 1 entries in  (i.e., the number of edges in the graph). If we now let , assume that  grows proportionally with , and that the subset sizes remain bounded by , then . This means that when comparing graphs  with equal numbers of edges the dominant terms of  are equal, since  and the comparison will depend on the remaining dominant term(14)

We let  be the set of all -dimensional vectors of nonnegative integers.

*Definition 3*. 

A mapping ,  is called stepwise monotone when the following holds. Let  be any element of  with at least two nonzero elements. Let  be any two components of  for which . Then let  be equal to , except that  and . Then , and  is called strictly stepwise monotone when the inequality can be replaced with strict inequality.

Note that  is a function of the vector of subset sizes . The stepwise operation described in Definition 3 generates a hierarchy of subset lists based on the tendency to concentrate larger subset sizes in fewer subsets. In terms of graphs, the ranking will be based on the tendency for a fixed number of edges to target a smaller number of nodes. We now show that  is strictly stepwise monotone. 

Lemma 3.

The mapping , interpreted as a function of the row totals  of , is strictly stepwise monotone.

Proof.

Noting the form of  in (9) it is convenient to write . Then from (14) we have (15)

Let  and  be two vectors from  as described in Definition 3. If , the ratio of the product in the second term of (15) may be written as (16)

Consider the quantities  and , where  are any integers for which . Then , and , from which it follows . Using (16) this inequality may be applied directly to the second term of (15) to verify the lemma. If , then we have the corresponding ratio: (17)

which can easily be shown to be less than one for all . The lemma therefore holds for this case as well.

**Figure 1:**
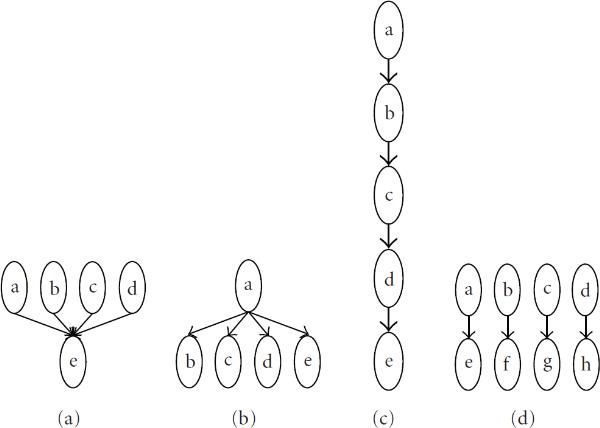
**Four sample graphs, each of 4 edges**.

Consider four graphs of  nodes consisting of  edges contained in the subgraphs in Figure 1. Denote the respective adjacency matrices by . It is easily verified that . This leads to two problems. First, we would like  and  to be scored equally. Second, graph () clearly has more interesting structure than (), but it has the same score. To address the first problem, we may score the transpose of the adjacency matrices, which gives . 

The second problem can be addressed using the modified score . We have, for fixed ,(18)

Thus, for large enough ,  and , so that  will be smaller for  and . Similarly , so that  can be seen to be sensitive to chain structure, whereas  is not. We then adopt the compound score:(19)

We omit from  a prefix consisting of a fixed number of bits which will indicate which score was the minimum.

## 4. Examples

In this section we apply Algorithm A to a set of examples, first a synthetic network based on a typical pathway, then one based on yeast genome perturbation experiments.

### 4.1. Synthetic Network (MAP Kinase)

**Figure 2:**
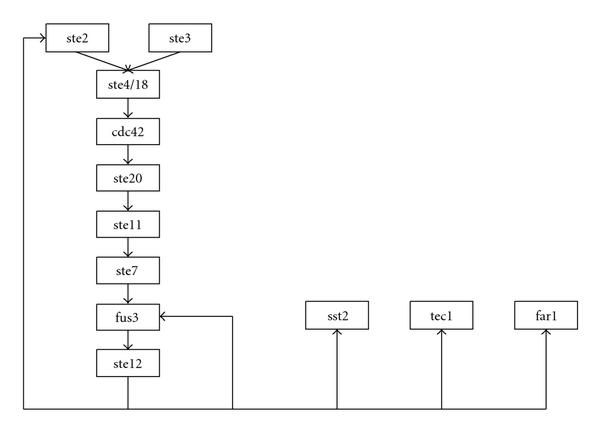
**Sample MAP Kinase network**.

The pathway illustrated in Figure 2 represents a known MAP kinase signal transduction cascade, used in [6] to illustrate a network model. This pathway possesses 12 genes and 13 edges. We will add  spurious genes to the model, allowing  to vary. The objective is to simulate a data matrix  as defined in steps (S1)-(S2), which might plausibly summarize experiments generated by this network. The strategy will be to first demonstrate the methodology on a statistically favorable case to clarify the objectives. The case will then be modified to present a scenario in which statistical noise plays a more prominent role.

#### 4.1.1. Model Simulation

Let  be the adjacency matrix of the graph in Figure 2. Gene  directly regulates  if . We also expect perturbation of  to affect genes further downstream; so we say that  and  are in an *order**relationship* if there is a path from  to  of  edges, and no shorter path exists. This holds if the th element of the product  is nonzero for  and zero for . 

If  and  are in an *order**relationship* simulate a normal random variable with mean  and variance 1, then let  be the -value associated with a hypothesis test  against . If  and  have no relationship, let  be uniformly distributed. 

A model is defined by characteristics  and . To study a given model the data matrix  is replicated 2500 times as described above. For each replication we apply Algorithm A, setting , . The compound score  of (19) is used. We use the elementwise exchangeable null hypothesis .

#### 4.1.2. Algorithm Evaluation

A study of the algorithm must take into account its dual purpose. We may accept  as an estimated accessibility graph which can be compared to the true graph. On the other hand, viewed as a multiple testing procedure, the objective is an efficient choice of  along a type of error curve, giving the expected number of true edges as a function of the total number of edges within graphs of the sequence . The properties of the error curve define the accuracy with which a cellular network can be inferred. Ideally, the error curve increases with slope 1 until the graph is constructed and then remains constant. Statistical variation forces deviation from this ideal; so the goal in the selection of  is to identify a position along the error curve such that below (or above) this position most new edges are true (or false) positives. 

We now discuss the calculation of the error curve. It will be convenient to restrict attention to relationships up to an order . Suppose that  is the true order  graph, in the sense that it contains edge  if and only if  and  have an order  relationship. In our example,  is equivalent to the graph in Figure 2. Let  represent a simulated replicate from the given model, from which we construct sequence . Let  be the number of edges of  contained in element  of . We will estimate two forms of the error curve. For the first, using replicates of  we calculate the sample mean value  of  for each . For the second, for each replicate  we use the edge value  minimizing , thus identifying , then capturing the pairs  to be displayed in the form of a scatter plot.

#### 4.1.3. Model 1 (Direct Regulation Only)

**Figure 3:**
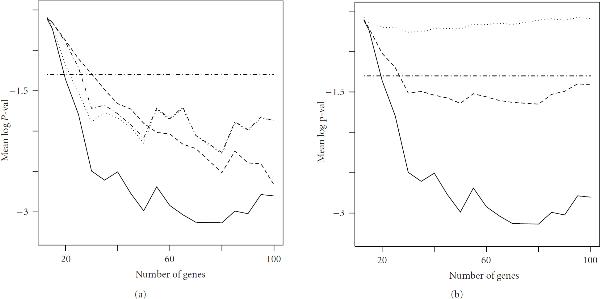
**Global significance plots for simulation study**. Number of genes  is varied within each plot. Probabilities are given on a base 10 logarithmic scale. A  axis is indicated. (a): Case  using statistics  [];  [];  [];  []. (b): Cases  [];  [];  [] using statistic .

We will first consider a simplified version of the problem, in which order  regulatory relationships are ignored ( for ). We study models defined by  and  in increments of 5. Each test network is replicated 2500 times. Figure 3 summarizes the achieved global significance for the statistics proposed in (8). In Plot 1 the four statistics proposed in (8) are applied to the models defined by  over the proposed range of . The plot shows the average attained global significance levels on a log scale (base 10). The horizontal axis represents a significance level of 0.05. Noticeably greater power is demonstrated for statistic , and significance levels are well below the 0.05 value over most of the range of , demonstrating the ability of the procedure to detect overall network structure. Sensitivity to the strength of the statistical evidence is demonstrated in Figure 3, Plot 2, in which average significance levels for models  over the proposed range of  are reported. Here we use only statistic , which will become our default choice. 

An interesting feature of these plots is the increase in power with the increase in the number of spurious genes. This is the opposite of what is usually expected in gene discovery but follows from the use of graphical structure as statistical evidence. The existence of such structure implies higher connectivity of a smaller subset of genes than would occur at random. The existence of a larger pool of unconnected genes should, to some extent, contribute to the significance of graphical discovery, since the existing structure would be less likely to have occurred by chance. Of course, the competing effect of false positives usually associated with multiple hypothesis testing will also exist. The relative importance of these effects remains to be analyzed.

**Figure 4:**
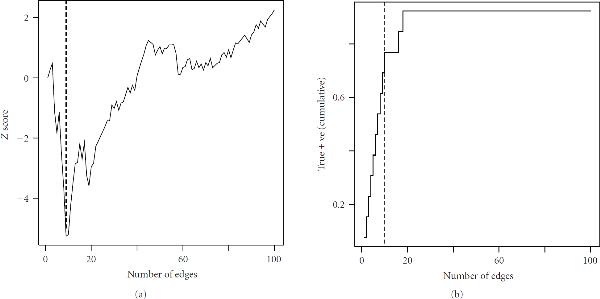
**Properties of sample data set , for  genes, **. (a): Value of -score sequence . Minimum point is located at edge 9, indicated by dashed line. (b): Cumulative proportion of true positive edges within sequence . There are 13 true edges. Minimum point of  (Plot 1) is indicated by dashed line.

A single simulation will illustrate the implementation of the algorithm. Here we assume  candidate genes, with effect size defined by . The -score values  are shown in Plot 1 of Figure 4. A clear minimum point is evident at 9 edges. Plot 2 shows the cumulative proportion of true positives among the edges defining the graphs in the sequence  (i.e., the number of true edges in  by edge). The minimum point at the 9th edge is clearly a point at which the graph is almost completely constructed, and above which most new edges will be false positives.

#### 4.1.4. Model 2 (Including Transitive Regulation)

**Figure 5:**
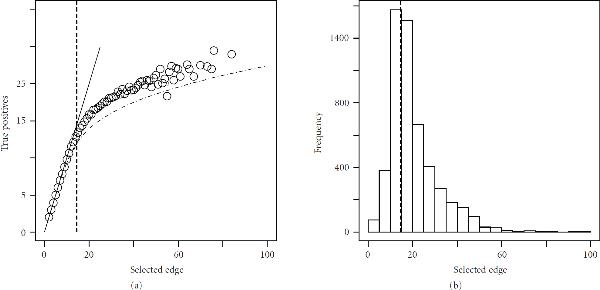
**Sample data set , for  genes, **. (a): error curve calculated by mean true positive  [], and scatter plot of selected minimum points and true positives . We use . The dashed line indicates the mode in Plot 2. (b): Histogram of selected minimum points . The mode is indicated by the dashed line.

We next include evidence of transitive regulations by simulating perturbation effects of size , with  for . We will examine specifically the  gene model and use 5000 replicates. The error curve is shown in Figure 5 (Plot 1) for up to order  relationships. Note that the error curve based on selected minimum points yields slightly higher values. We conjecture that the minimum selection process introduces greater accuracy (discussion in the next subsection pertains to this issue). As expected, both error curves are at first of unit slope, up until a point at which false positives begin to dominate. As emphasized earlier, the error curve represents an inherent limit of the accuracy possible under given experimental conditions. The role of our procedure is therefore to determine a suitable location along that curve. In Figure 5 (Plot 2), a histogram of the captured minimum points  is shown. Interestingly, the mode of the histogram is located precisely where the error curve is no longer of unit slope, which is the point we wish to identify.

### 4.2. Yeast Genome Expression Data

In [19] a series of gene deletion and drug experiments are reported, resulting in a compendium of 300 microarray gene expression profiles on the yeast genome. We extracted 266 genes for which single deletion experiments were performed. By matching the responses for those genes a  data matrix  of perturbation effect -values was constructed (the -values used are those reported in [19]). Algorithm A was applied using a maximum of  edges, using  replications of a null matrix; then  was calculated as above. These replications were supplemented by an application with settings , . We use the element wise exchangeable null hypothesis .

**Figure 6:**
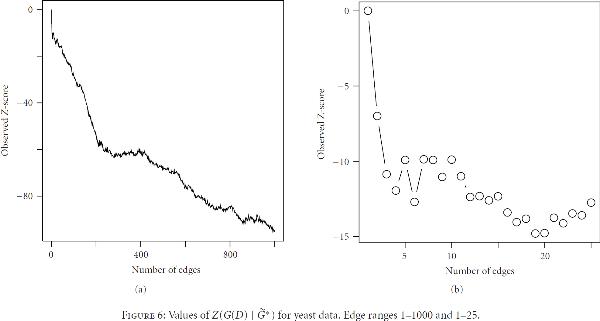
**Values of  for yeast data**. Edge ranges 1–1000 and 1–25.

The -scores are shown in Figure 6. A significant deviation from zero is shown almost immediately and persists throughout the observed range. The global -value at 1-2 edges is estimated as  and decreases rapidly beyond this point. Table 1 lists the edges associated with the 10 lowest -values in . At this point obvious graph structure is apparent, as the first four edges all have a common parent *tup1*. It is interesting to note that the -score falls as edges 2 to 4 are added, each of which contains a gene found in the previous edges. Edge 5, however, introduces two new genes, at which point the -score increases. In fact, this rule persists up to the 12th edge; that is, the -score decreases if and only if at least one gene of a new edge exists among the previous edges. It also holds among 83%, 74%, and 63% of the first 25, 100, and 1000 edges, respectively. 

**Table 1:** Graph edges associated with 10 lowest ranking -values for yeast data

-val	Response	Perturbed	-val	Response	Perturbed
Rank	Gene	Gene	Rank	Gene	Gene
1	hpa3	tup1	6	yer066c-a	tup1
2	yor009w	tup1	7	rts1	yor015w
3	pau2	tup1	8	yhr022c	ssn6
4	yhr022c	tup1	9	phd1	tup1
5	ste2	ste12	10	ald5	yer050c

**Figure 7:**
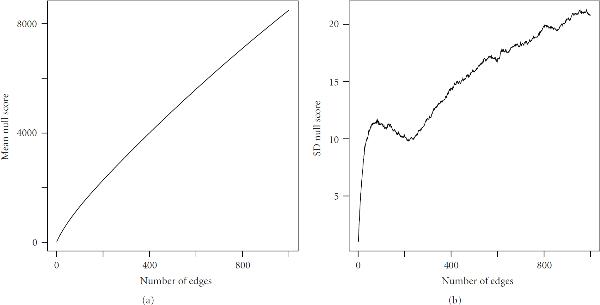
Mean and standard deviation of graph scores for  null perturbation matrix by edge number.

In this example, the -score is clearly able to distinguish between edges which contribute to graph structure and those that do not up to some number of edges. The application of this principle over a large range of edges is complicated by the increasingly complex statistical properties of the graph score, as suggested in Figure 7. While the mean score increases smoothly, the growth of the standard deviation is more complicated. We would expect this to some degree. The number of offspring for each node would be approximately Poisson distributed when such numbers are small, but eventually this probability law will no longer hold when subsets become large enough. It is therefore problematic to identify interesting features of the -score plot over large ranges of edge number.

**Figure 8:**
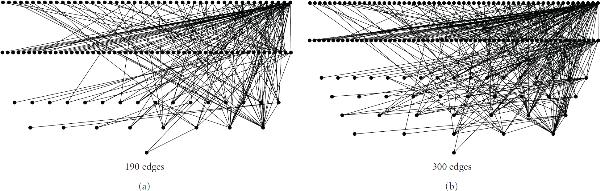
**Estimated accessibility graphs for yeast genome data, using 190 and 300 edges**. The choice of 190 genes represents the application of the Benjamini-Hochberg FDR control procedure.

Finally, we make a note comparing multiple testing procedures (MTPs) and our proposed graph-based procedure. Accepting the -values reported in [19], two well-known MTPs were applied to the -values of matrix  (see [1] for details). Using the Bonferroni procedure (FWER = 0.05) 41 -values are rejected, whereas using the Benjamini-Hochberg procedure (FDR = 0.05) 190 -values are rejected. Figure 8 displays the connectivity graphs formed from the first 190 and the first 300 edges. All edges point "downward" in the diagram (arrows are omitted for clarity). Three exceptions are indicated by dashed lines, which are bidirectional. The graphs contain no cyclic behavior other than these edges (a simple simulation experiment confirms that this level of cyclicity is compatible with the Erdös-Renyi random graph model). 

If we accept the 190 edge graph as that resulting from the application of an MTP, we then note that the proposed graph-based method results in significantly more structural discovery. The global significance level for a graph with 1000 edges can be taken as extremely small from an estimated -score of −95.4. This significance level applies to subgraphs from the sequence . Similarly, additional structure in the 300 gene graph compared to the 190 gene graph can be clearly seen. In order to define a "highly connected gene," we simulate random graphs to estimate a distribution of a gene's edge order . For  and  edges among 266 nodes we have  and . Thus, we define any gene with at least 4 and 5 edges as "highly connected" in the respective graphs. Under these criteria, the respective graphs contain 33 and 43 such genes. The most connected gene in the 190 gene graph is  with 38 edges. This gene is also the most connected gene in the 300 gene graph (46 edges). In general, more highly connected genes are added between edges 190 and 300, while additional edges are added to already highly connected genes.

## 5. Conclusion

A common problem in the statistical analysis of high-throughput data is the selection of a threshold for statistical evidence which controls false discovery. Such data is often used to construct graphical models of gene interactions. A threshold selection procedure was proposed which is based on the observed graphical structure implied by a given threshold. This procedure can be used both for threshold selection and to estimate a global significance level for graphical structure. The method was demonstrated on a small simulated network as well as on the "Rosetta Compendium" [19] of yeast genome expression profiles. The methodology proved to be accurate and computationally feasible.

Further investigation is warranted in a number of issues. The graphs investigated here were unconstrained directed graphs. Application to undirected graphs and directed acyclic graphs (DAGs) will require more sophisticated graph simulation algorithms. Additionally, the long range statistical behavior of the proposed graph code is complex. Such issues will need to be carefully examined before a general threshold selection technique can be proposed.

A software implementation of the proposed procedures is available from the author's web site, in the form of an R library at http://www.urmc.rochester.edu/biostat/people/faculty/almudevar.cfm.
